# Supplementary material for: Understanding coordination reaction for producing stable electrode with various low work functions
Source: Nat Commun. 2020 Jul 24;11:3700. doi: 10.1038/s41467-020-17548-z (PMC7382499; doi:10.1038/s41467-020-17548-z)
Supplement: Supplementary file 1 — Supplementary information [file 41467_2020_17548_MOESM1_ESM.pdf]

Supplementary Information

Understanding coordination reaction for producing stable electrode with various low work functions

Hirohiko Fukagawa[1,*], Kazuma Suzuki[1], Hirokazu Ito[2], Kaito Inagaki[2], Tsubasa Sasaki[1], Taku Oono[1], Munehiro Hasegawa[3], Katsuyuki Morii[3,4] and Takahisa Shimizu[1]

[1] Japan Broadcasting Corporation (NHK), Science & Technology Research Laboratories, 1-10-11 Kinuta, Setagaya-ku, Tokyo 157-8510, Japan
[2] Tokyo University of Science, 1–3 Kagurazaka, Tokyo 162-8610, Japan
[3] Nippon Shokubai Co., Ltd., 5-8 Nishi Otabi-cho, Suita, Osaka, 564-8512, Japan
[4] Nippon Shokubai Research Alliance Laboratories, Osaka Univ., Osaka, 565-0871, Japan

*Corresponding author: fukagawa.h-fe@nhk.or.jp

Supplementary Figures

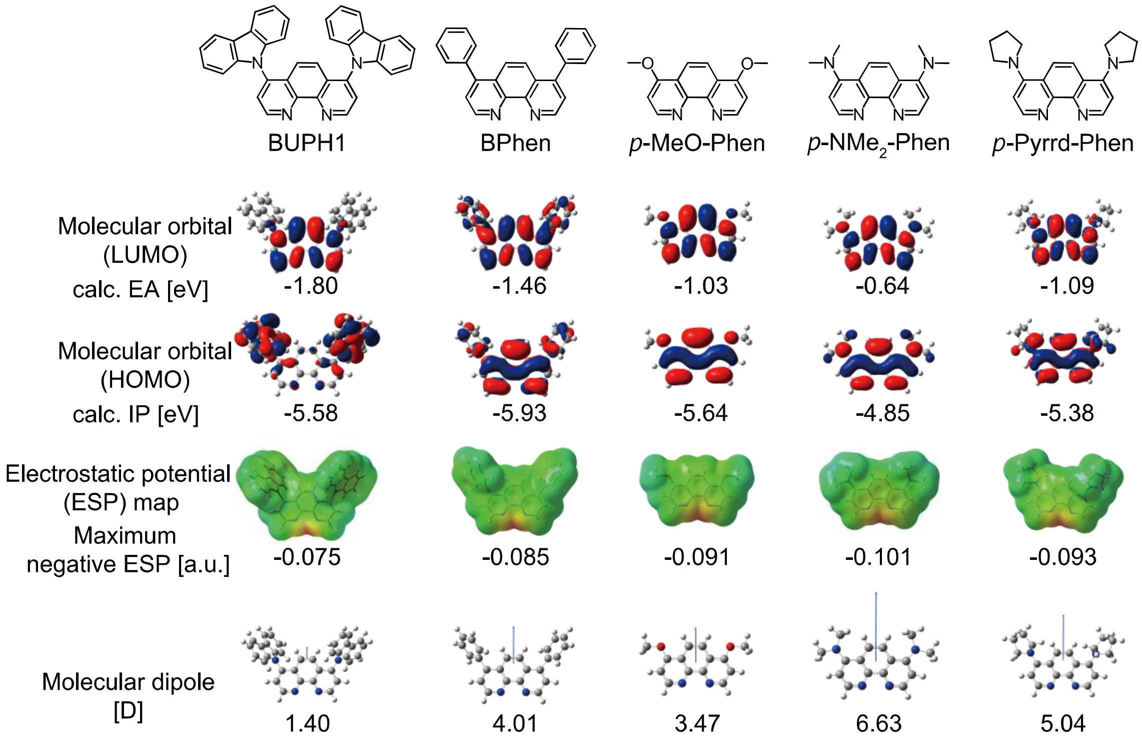

Supplementary Figure 1 | DFT calculation results for five Phen derivatives.

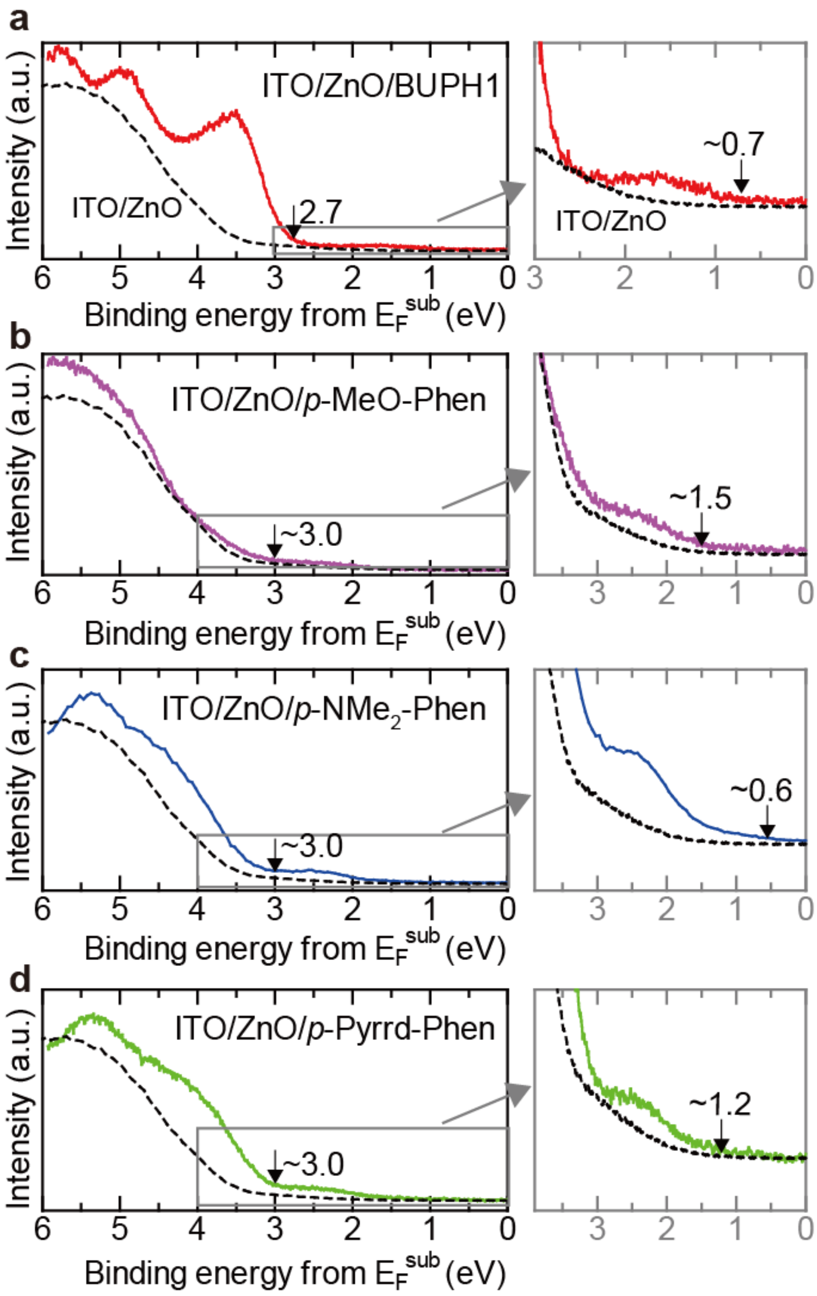

Supplementary Figure 2 | HeI UPS spectrum of each film. HeI UPS spectra of (**a**) ITO/ZnO/BUPH1, (**b**) ITO/ZnO/*p*-MeO-Phen, (**c**) ITO/ZnO/*p*-NMe_2-Phen and (**d**) ITO/ZnO/*p*-Pyrrd-Phen. The position of the gap state is also shown[1].

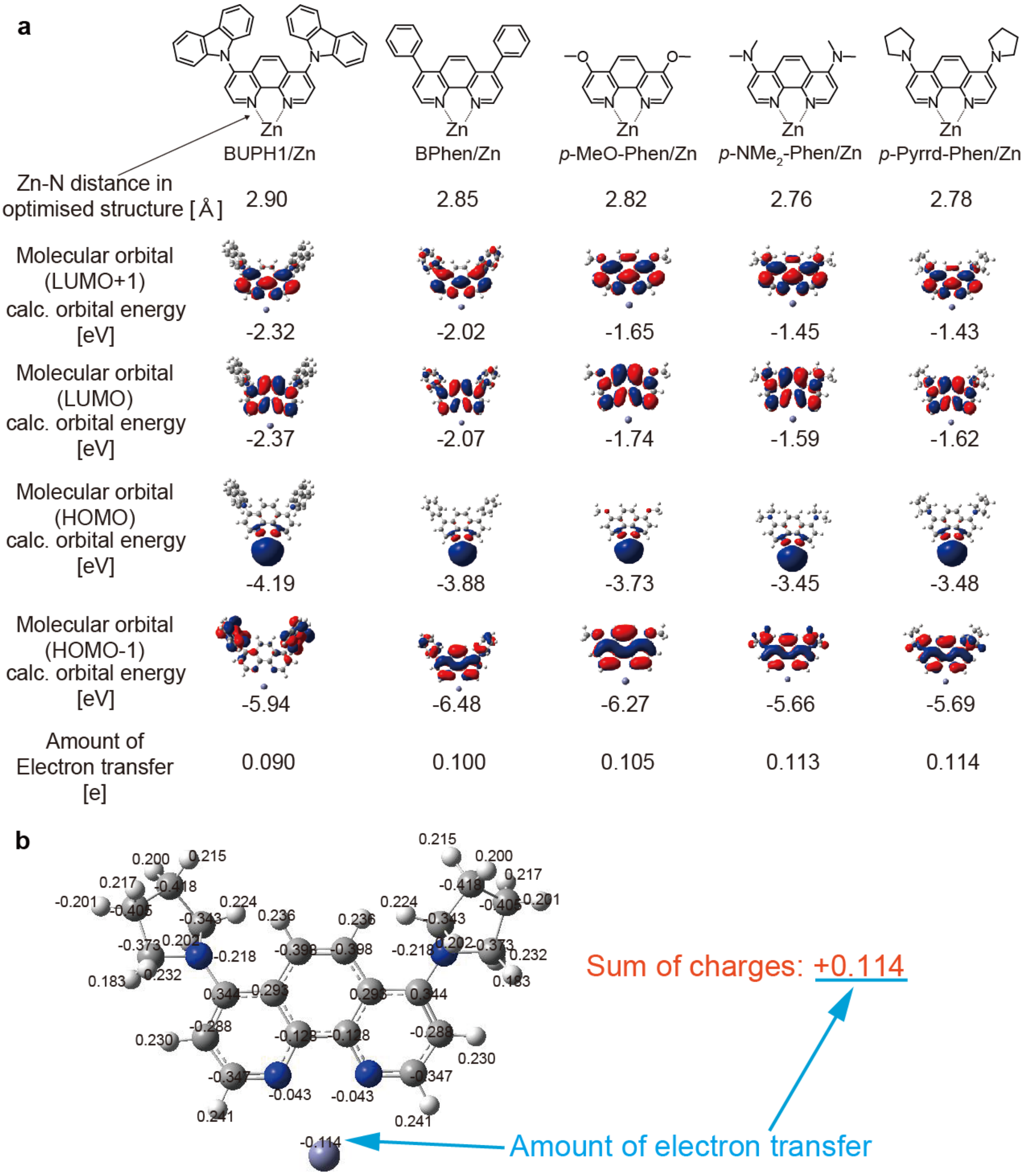

Supplementary Figure 3 | DFT calculation results for five Phen derivatives with Zn. (**a**) Distance between N in the Phen derivative and Zn in the optimised structure, the calculated molecular orbitals, the calculated orbital energies and the calculated amount of electron transfer. (**b**) Schematic of atomic charge distribution (Mulliken) for *p*-Pyrrd-Phen with Zn system estimated by DFT calculation.

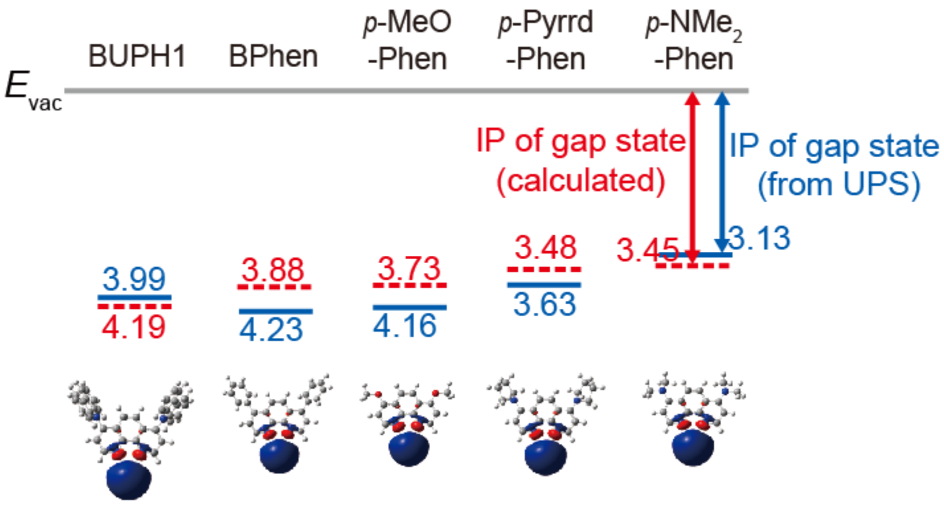

Supplementary Figure 4 | Schematic illustrations of the IPs of gap states.

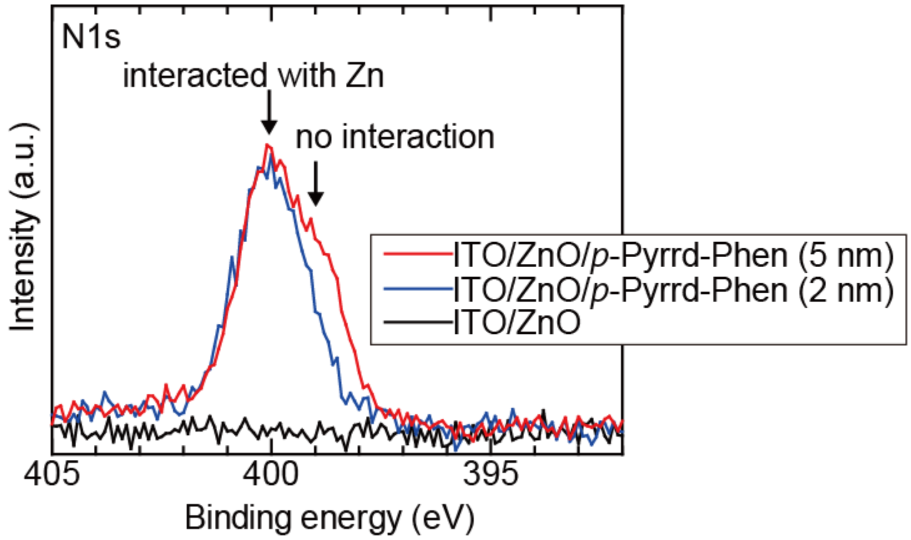

Supplementary Figure 5 | X-ray photoelectron spectroscopy spectra of *p*-Pyrrd-Phen on ITO/ZnO with different thicknesses.

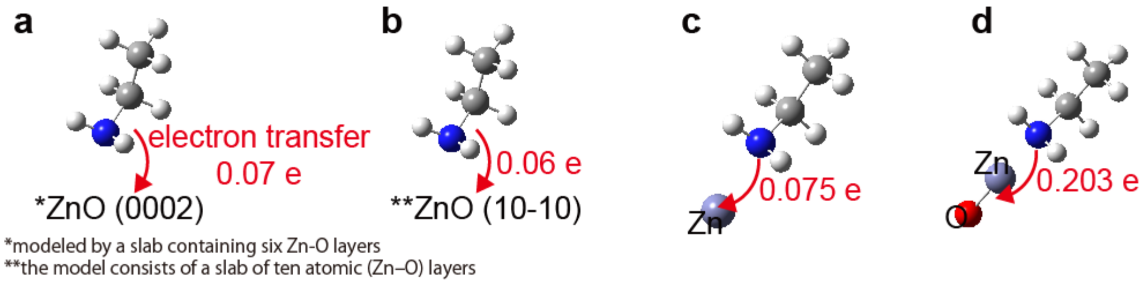

Supplementary Figure 6 | Schematic illustrations of the calculated amount of electron transfer (ET) from ethylamine to metallic counterparts. The metallic counterparts used for calculation are (**a**) ZnO (0002)[2], (**b**) ZnO (0002)[2], (**c**) Zn atom and (**d**) ZnO.

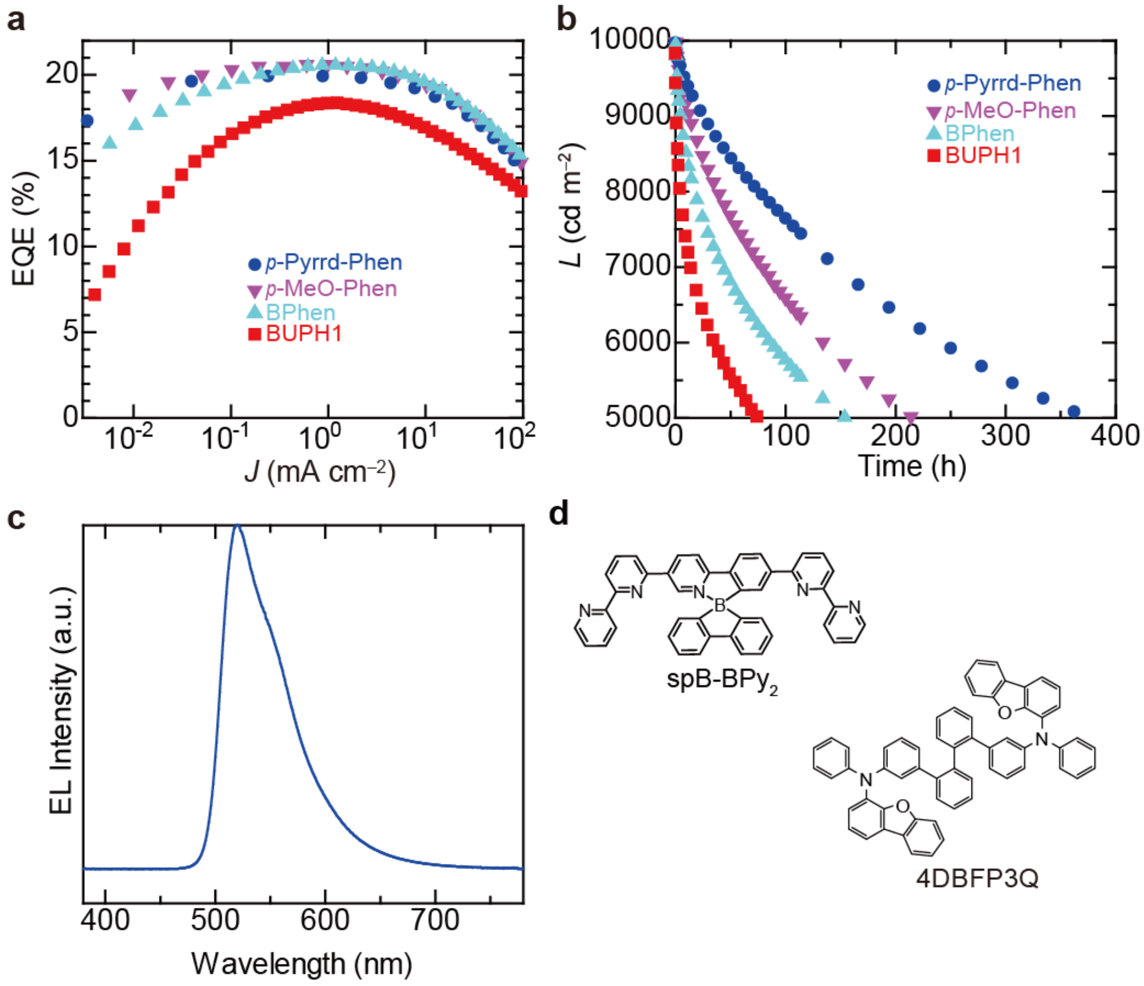

Supplementary Figure 7 | Characteristics of cOLEDs employing Phen derivatives as EIL. (**a**) EQE–current density (*J*) curves of cOLEDs. (**b**) Luminance–time characteristics of devices under a constant dc. (**c**) EL spectrum of cOLED employing *p*-Pyrrd-Phen as EIL. (**d**) Chemical structure of the materials used in the cOLEDs.

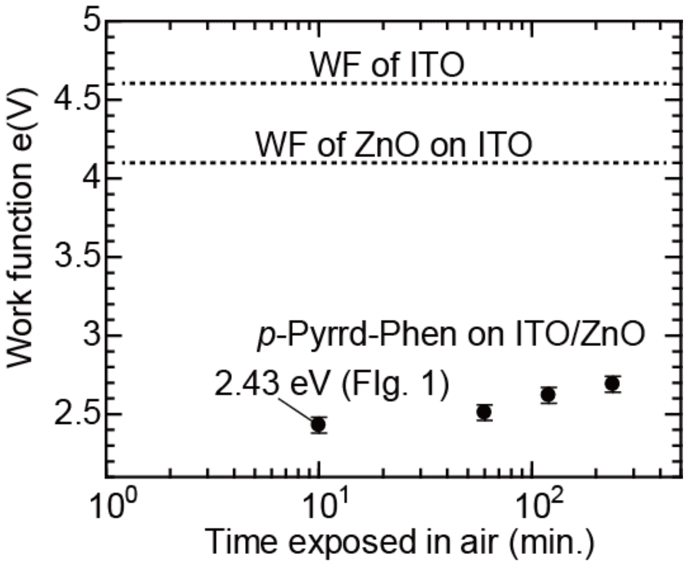

Supplementary Figure 8 | Evolution of the work function of *p*-Pyrrd-Phen on ITO/ZnO for different air exposure times. As in Ref. 2, the effect of air exposure on WF is small, especially for an exposure time within 60 min.

Supplementary Table

Supplementary Table 1 | Summary of differences in device configurations/characteristics between this work and previous work.

	Colour of emitter	EIL	Voltage at current density of 10 mA/cm^2	Applicability to the production line[4]
Previous work[3]	Red	Ag-doped *p*-MeO-Phen	3.3 V	Not easy (due to the Ag-doping)
cOLED using *p*-MeO-Phen	Green	*p*-MeO-Phen[3]	6.8 V	Easy
cOLED using *p*-Pyrrd-Phen	Green	*p*-Pyrrd-Phen (this work)	4.9 V	Easy
cOLED using *p*-Pyrrd-Phen	Green	LiF	5.2 V	Easy

Supplementary Methods

Materials:

OLED materials were mainly purchased from Luminescence Technology Corporation (Taiwan) and were used after sublimation. *p*-MeO-Phen was purchased from Sigma-Aldrich. Polyethyleneimine [PEI, used in the inverted OLED in Fig. 3] was supplied by Nippon Shokubai. Both **4,7-Dimethylamino-1,10-phenanthroline** (*p*-NMe2-Phen) and **4,7-Pyrrolidinyl-1,10-phenanthroline** (*p*-Pyrrd-Phen) were synthesised following the simple procedure described below:

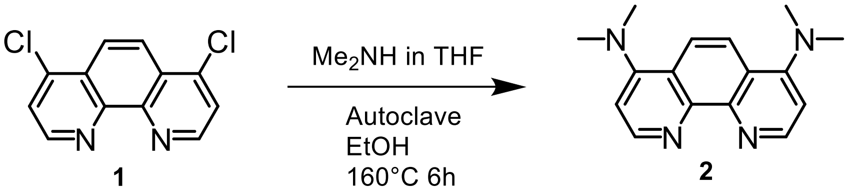

4,7-Dimethylamino-1,10-phenanthroline (*p*-NMe2-Phen)**:**

4,7-Dichloro-1,10-phenanthroline (**1**, 2.0 g, 8.03 mmol,1.0 eq.), dimethylamine (2.0 M in THF) (80 mL, 12.0 mmol, 25 eq) and ethanol (40 mL) were added to a 300 mL autoclave. The suspension was heated and stirred at a bath temperature of 160 °C for 5 hours. After cooling, the reaction solution was concentrated, and 0.5 M aqueous solutions of sodium hydroxide (50 mL) and ethyl acetate (50 mL) were added to the residue, followed by stirring, and the reaction solution was separated into two layers. The aqueous layer was extracted with ethyl acetate (50 mL), and the combined organic layers were washed successively with city water and saturated brine, dried over anhydrous sodium sulfate, and concentrated to give a brown solid (2.75 g). Column purification using chloroform gave a beige solid (1.92 g). Acetone (13 mL) was added to the solid, and the solid was dissolved by heating at a bath temperature of 40 °C. Diethyl ether (15 mL) was added to this solution and recrystallised. Dispersion and washing with ethyl ether, filtration, washing with ethanol cooled to −30° C and drying under high vacuum at 50 °C for 8 hours gave a light brown powder of *p*-NMe2-Phen (1.02 g, 3.83 mmol, 45%). The purity of the material we used in the experiment was estimated to be 98.95% from the results of high-performance liquid chromatography (HPLC).

^{1}H NMR (600MHz, $CDCl_3$) δ 3.07 (s, 12 H) 7.01 (d, *J*=5.28 Hz, 2 H) 7.27 (s, 2 H) 7.97 (s, 2 H) 8.91 (d, *J*=4.99 Hz, 2 H)

^{13}C NMR (100.4MHz, $CDCl_3$) δ=157.3, 149.7, 121.7, 120.5, 109.3, 43.7

Equipment：Shimadzu HPLC (Prominence)

Column：L-column 2 ODS

Eluent：Acetonitrile/ H_2O in 0.015M KH_2PO_4= 30/70

Column temp.:40 °C

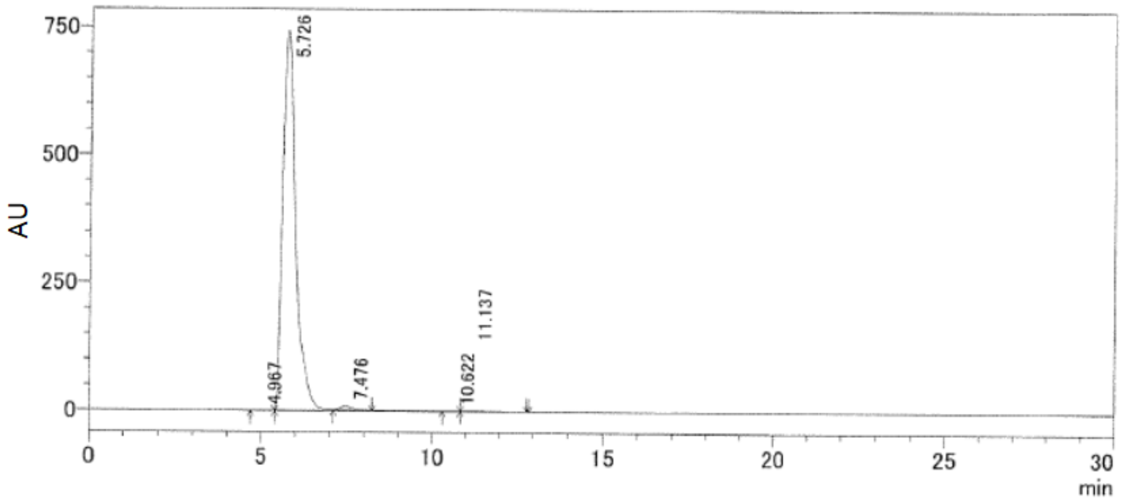

Retention time (min)	Area %
4.967	0.019
5.726	98.951
7.476	0.930
10.622	0.021
11.137	0.079

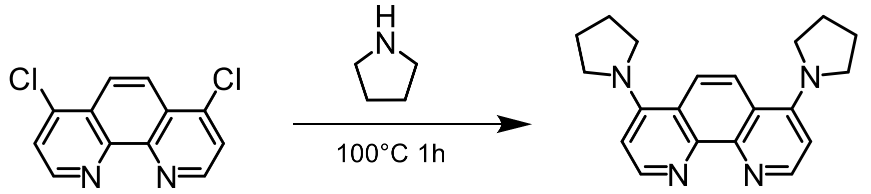

1 2

4,7-Pyrrolidinyl-1,10-phenanthroline (*p*-Pyrrd-Phen)**:**

A mixture of 4,7-Dichloro-1,10-phenanthroline (**1**, 3.0 g) and pyrrolidine (19.5 mL) was placed in a 100 mL flask and heated to reflux in a 100 °C oil bath for 1 hour. The mixture was allowed to cool to room temperature then concentrated in vacuo, water was added thereto, and the precipitated solid was collected by filtration and sonication. The resulting solid was dried in vacuo and dissolved in 100 ml of MeOH. Activated carbon was added to the mixture, the mixture was stirred at room temperature for 1 hour, and then the insoluble substance was filtered off. The filtrate was concentrated in vacuo and the resulting solid was recrystallised from 9 ml of MeOH. The obtained solid was washed with a small amount of MeOH and dried under reduced pressure to obtain compound **2** (*p*-Pyrrd-Phen ,1.69g, 44%) as a white solid. The purity of the material we used in the experiment was estimated to be 99.03% from the results of Ultra Performance LC (UPLC).

^{1}H NMR (600MHz, $CDCl_3$) δ 2.05 (dt, *J*=6.16, 3.37 Hz, 8 H) 3.65 - 3.72 (m, 8 H) 6.70 (d, *J*=5.58 Hz, 2 H) 7.94 (s, 2 H) 8.73 (d, *J*=5.58 Hz, 2 H).

^{13}C NMR (600MHz, $CDCl_3$) δ .25.94, 52.20, 105.43, 119.31, 119.39, 148.31, 149.20, 152.77.

Equipment：Waters ACQUITY UPLC

Column：BEH C18 1.7 µm ø2.5 mm*50 mm

Eluent：Acetonitrile/0.1% TFA in H2O = 50/50

Column temp.:40 °C

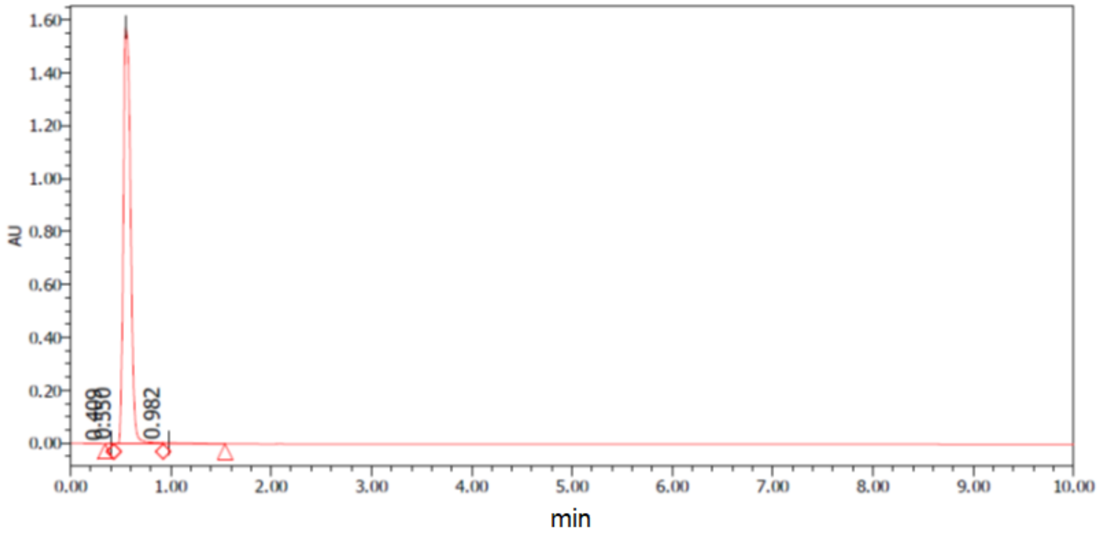

Retention time (min)	Area %
0.409	0.10
0.550	99.03
0.982	0.87

Supplementary References

1. Yoshida, H. Electron Transport in Bathocuproine Interlayer in Organic Semiconductor Devices. *J. Phys. Chem. C* **119**, 24459-24464 (2015).
2. Zhou, Y. *et al.* A universal method to produce low-work function electrodes for organic electronics. *Science* **336**, 327-332 (2012).
3. Bin, Z. *et al.* Making silver a stronger n-dopant than cesium via in situ coordination reaction for organic electronics. *Nat. Commun.* **10**, 866 (2019).
4. Eritt, M. *et al.* OLED manufacturing for large area lighting applications. *Thin Solid Films* **518**, 3042–3045 (2010).
